# Supplementary material for: Nanopore sequencing enables near-complete de novo assembly of Saccharomyces cerevisiae reference strain CEN.PK113-7D
Source: FEMS Yeast Res. 2017 Sep 13;17(7):fox074. doi: 10.1093/femsyr/fox074 (PMC5812507; doi:10.1093/femsyr/fox074)
Supplement: Supplemental material — Supplementary data are available at FEMSYR online. [file fox074_supp.zip › Additional File 1 Pilon correction benchmark.docx]

# Additional File 1

## Benchmark of short read polishing methods using Pilon

Polishing with Illumina data further improves the quality of assembly primarily by reducing the number of homopolymer errors in the nanopore assembly. Using Pilon (Walker *et al.* 2014), this polishing step can be done with or without correction of predicted structural variations. Although information on structural variation should be captured more effectively in long reads than in short read data, repetitive sequences (including homopolymer repeats) can easily reach more than 10 nucleotides and these corrections would be flagged by Pilon as structural variants instead of short indels. While polishing may correct long repetitive sequence errors, sequence gaps and misassemblies can also be introduced by Pilon; affecting coding sequences such as paralogous genes and genes with copy number variation. We therefore benchmark Pilon’s polishing step with and without structural variant correction based on the subsequent accuracy of ORF predicted. We used a 150 bp paired-end Illumina library with 161x coverage. For short sequence correction, we used the (--fix bases parameter) using only reads with a minimum mapping quality of 20 (minmq 20 parameter). We used default parameters for indel and structural variant correction.

Structural variant correction led to the loss of several ORFs. When allowing for structural variation, a total of 6,307 nucleotides involving structural variants of at least 10 nt were corrected—241 were removed and 6,066 inserted. The structural corrections involved repetitive sequences including long-stretches of homopolymer-like repeats. Structural variant correction resulted in the loss of 12 ORFs, the addition of 4 ORFs, and a different annotation for 8 ORFs when compared to short-sequence-only correction. While none of the additional ORFs could be confirmed from external data, many lost ORFs such as YBR122C were shown to be present in previous assemblies of the CEN.PK113-7D genome (Nijkamp *et al.* 2012). Therefore we decided to polish using only SNP and short indels correction despite the inability to resolve long homopolymer repeats. This resulted in the correction of 21,552 nucleotides (17,949 bp added and 3,603 bp removed).
