# Supplementary material for: Nanopore sequencing enables near-complete de novo assembly of Saccharomyces cerevisiae reference strain CEN.PK113-7D
Source: FEMS Yeast Res. 2017 Sep 13;17(7):fox074. doi: 10.1093/femsyr/fox074 (PMC5812507; doi:10.1093/femsyr/fox074)
Supplement: Supplemental material — Supplementary data are available at FEMSYR online. [file fox074_supp.zip › Additional File 5 Chef Gel analysis.docx]

**Additional File 5: Analysis by CHEF and southern blotting with a *MAL* probe of various CEN.PK113-7D stocks provides evidence of the presence of a translocation between chromosomes III and VIII only in CEN.PK113-7D Delft and confirms chromosome structure heterogeneity in this stock.**

## **Materials and methods**

Yeast strains

The *Saccharomyces cerevisiae* strains CEN.PK113-7D, CEN.PK113-5A, CEN.PK113-5D and CEN.PK122 used in this study are derived from the CEN.PK strain family (Entian and Kötter 2007, Nijkamp *et al.* 2012). The genotype and origin of all strains used in this study is shown in Table 1. Strains provided by different laboratories were grown once until stationary phase in liquid YPD medium (containing 10 g/l yeast extract, 20 g/l peptone and 20 g/l glucose) upon arrival and 1 mL aliquots were made which were stored at -80˚C since. Genomic DNA of CEN.PK113-7D Delft for PCR amplification of southern blotting probes was extracted using the YeaStar™ Genomic DNA Kit (Zymo Research Corporation, Irvine, CA) according to the manufacturer’s instructions from stationary phase cultures grown on liquid YPD medium at 30˚C and 200 rpm.

In order to investigate the possibility of a heterogeneous population in CEN.PK113-7D Delft, single colony isolates were made using Fluorescence-Assisted Cell Sorting (FACS). To this end CEN.PK113-7D Delft was grown overnight in YPD at 30˚C and 200 rpm. Single cells were sorted from this culture onto a 96-well plate with 200 µL/well of YPD supplemented with 20 g/l agar using a FACS Aria II SORP (BD, Franklin Lakes, NJ) with a 70 µm nozzle and a single cell sorting mask (0/32/16). Gating of single cells was performed stringently on a FSC/SSC plot. The YPD plates were incubated overnight at 30˚C and four single colonies were stocked as CEN.PK113-7D SCI 1 to SCI 4.

**Table 1: Strains used throughout this study with their relevant genotype and their source.**

| **Strain** | **Relevant genotype** | **Source** |
| --- | --- | --- |
| CEN.PK113-5A | *MATa MAL2-8c trp1-289 leu2-3,112 his3-Δ1* | Kindly provided by Dr Kötter and Dr Entian, Goethe University Frankfurt (Germany) in 2007. |
| CEN.PK113-5D | *MATa ura3-52* | Kindly provided by Dr Kötter and Dr Entian, Goethe University Frankfurt (Germany) in 2004. |
| CEN.PK122 | *MATa/MATα MAL2-8c* | Kindly provided by Dr Kötter and Dr Entian, Goethe University Frankfurt (Germany) in 2013. |
| CEN.PK113-7D Delft | *MATa MAL2-8c* | Kindly provided by Dr Kötter and Dr Entian, Goethe University Frankfurt (Germany) in 2001. |
| CEN.PK113-7D Delft ALT | *MATa MAL2-8c* | Kindly provided by Dr Kötter and Dr Entian, Goethe University Frankfurt (Germany) in 2003. |
| CEN.PK113-7D Frankfurt | *MATa MAL2-8c* | Kindly provided by Dr Kötter and Dr Entian, Goethe University Frankfurt (Germany) in 2016. |
| CEN.PK113-7D Amyris | *MATa MAL2-8c* | Kindly provided by Amyris (Emeryville, CA) in 2016. |
| CEN.PK113-7D Chalmers | *MATa MAL2-8c* | Kindly provided by Dr. Siewers, Chalmers University of Technology (Göteborg, Sweden) in 2016. |
| CEN.PK113-7D Delft SCI 1 | *MATa MAL2-8c* | Single colony isolate made using fluorescence assisted cell sorting from CEN.PK113-7D Delft. |
| CEN.PK113-7D Delft SCI 2 | *MATa MAL2-8c* | Single colony isolate made using fluorescence assisted cell sorting from CEN.PK113-7D Delft. |
| CEN.PK113-7D Delft SCI 3 | *MATa MAL2-8c* | Single colony isolate made using fluorescence assisted cell sorting from CEN.PK113-7D Delft. |
| CEN.PK113-7D Delft SCI 4 | *MATa MAL2-8c* | Single colony isolate made using fluorescence assisted cell sorting from CEN.PK113-7D Delft. |

## Electrophoretic karyotyping and Southern blotting

Chromosomal DNA plugs with 1% CleanCut agarose for electrophoretic karyotyping were prepared according to the manufacturer’s instructions for yeast using the CHEF Genomic Plug Kit (Bio-Rad, Richmond, CA). For each mL of plug, about 6 x 10^8^ cells were used based on OD_660_ (60OD_660_/mL) for haploid strains and half this amount for diploid strains. 40µL Plugs were inserted into a 1% Megabase agarose (BioRad) gel and the chromosomes were separated in 0.5X TBE buffer (45 mM Tris, 45 mM H_3_BO_3_, 1 mM EDTA, pH 8.3) kept at 14˚C in a CHEF-DR II apparatus (Bio-Rad) with a voltage of 5 V/cm, a pulse angle of 120˚ and pulse time of 60 sec during 28 h followed by a pulse time of 90 sec for another 16 h. The gel was stained in 200 mL 0,5X TBE with 1 mg/mL ethidium bromide, de-stained in 200 mL 0,5X TBE and imaged under UV light. DNA from the gel was transferred to a membrane using the TurboBlotter Nytran SPC 0.45 µm, 15 × 20 cm Kit (GE Healthcare Europe GmbH, Diegem, Belgium) according to the manufacturer’s instructions during 2 h 15 min and fixated by exposure to a TUV 30 W T8l amp (Philips, Eindhoven, Netherlands) at a radiation peak of 253.7 nm during 2 min. Probes for the *MAL32* loci were PCR amplified from genomic DNA of CEN.PK113-7D Delft using DreamTaq PCR Master Mix (Thermo Scientific, Wilmington, DE) and primers mal32fw (GCAGAAGGGCAATCTTTGAAAGTG) and mal32rv (AGCAGCAAACAGCGTCTTGTC). The probes were labelled and hybridized to the membrane according to the manufacturer’s instructions using the AlkPhos Direct Labeling Module (GE Healthcare Europe GmbH). The chemiluminescent signal of the labelled probes bound to the membrane was produced by 5 min incubation with CDP-Star and captured/visualized on an Amersham Hyperfilm ECL (GE Healthcare Europe GmbH) during 15 min exposure. The film was developed using Carestream® Kodak® autoradiography GBX developer/replenisher for 1 min and fixer/replenisher for 2 min (Kodak, Rochester, NY, USA).

## **Results:**

To investigate whether this translocation might occur in various CENPK113-7D stocks and isolates, several strains were analysed by contour-clamped homogeneous electric field electrophoresis (CHEF). The proposed recombination between chromosomes III and VIII would involve a marked difference in the size of these chromosomes which should be detectable by CHEF. In addition to CEN.PK113-7D Delft, CEN.PK113-7D stocks from various locations were analysed, as well as related auxotrophic CEN.PK strains CEN.PK113-5D (*ura3-52*) and CEN.PK113-5A (*trp1-289*, *leu2-3*, *his3D1*) and the prototrophic diploid CEN.PK122 from Delft. For CEN.PK113-7D Delft, four single colony isolates were also karyotyped. While the other tested strains showed identical band patterns to CEN.PK113-7D Frankfurt, CEN.PK113-7D Delft SCI2 to SCI4 do not show a band for chromosome III (Figure 1, left, lanes 11-13). As chromosome III contains essential genes, this suggests presence of translocated chromosomes III-VIII and VIII-III. The presence of chromosomes VIII, III-VIII, VIII-III cannot be evaluated as they would be masked by the bands of similar size from chromosomes V, XI and I, respectively. In order to detect the potential recombined chromosome III-VIII and in order to settle an ongoing debate about the number of MAL loci in CEN.PK113-7D (Nijkamp *et al.* 2012), Southern blotting was performed with a *MALX2* probe targeting the highly conserved maltase genes of the MAL loci (>99% identity at DNA level). This probe could bind on *MAL12* on chromosome VII, *MAL22* on chromosome III, *MAL32* on chromosome II and the hypothesized *MAL42* on chromosome XI. Additionally the MAL32 could to a lesser extent also bind the IMA genes that exhibit sequence similarity ranging from 60 to 70% with the maltase probe. For all tested strains, no differences were noticeable for bands pattern of *MAL12*, *MAL32* and the IMA genes (Figure 1, right). Consistent with the CHEF results, no bands for *MAL22* were observed for chromosome III in three out the four cell sorted CEN.PK113-7D cell lines (Figure 1, right, lanes 11-13), suggesting the absence of chromosome III in those strains. The absence of *MAL22* on chromosome III was correlated with the presence of a band at the height of chromosome III-VIII. CEN.PK113-7D Delft showed both a band on chromosome III and on chromosome III-VIII (Figure 1, right, lane 1). These hybridization patterns suggest that the previously sequenced CEN.PK113-7D strain was a mixed culture of strains which all together harboured the four chromosome forms, III, VIII, III-VIII and VIII-III. This unfortunately led to the mis-annotation of MAL4 locus that was in fact the MAL2 locus on translocated chromosome III-VIII. Furthermore, as CEN.PK113-7D SCI1 to SCI4 have either a translocated or a non-translocated chromosome III, it seems that both architectures are relatively stable as they are maintained by the whole single colony population.


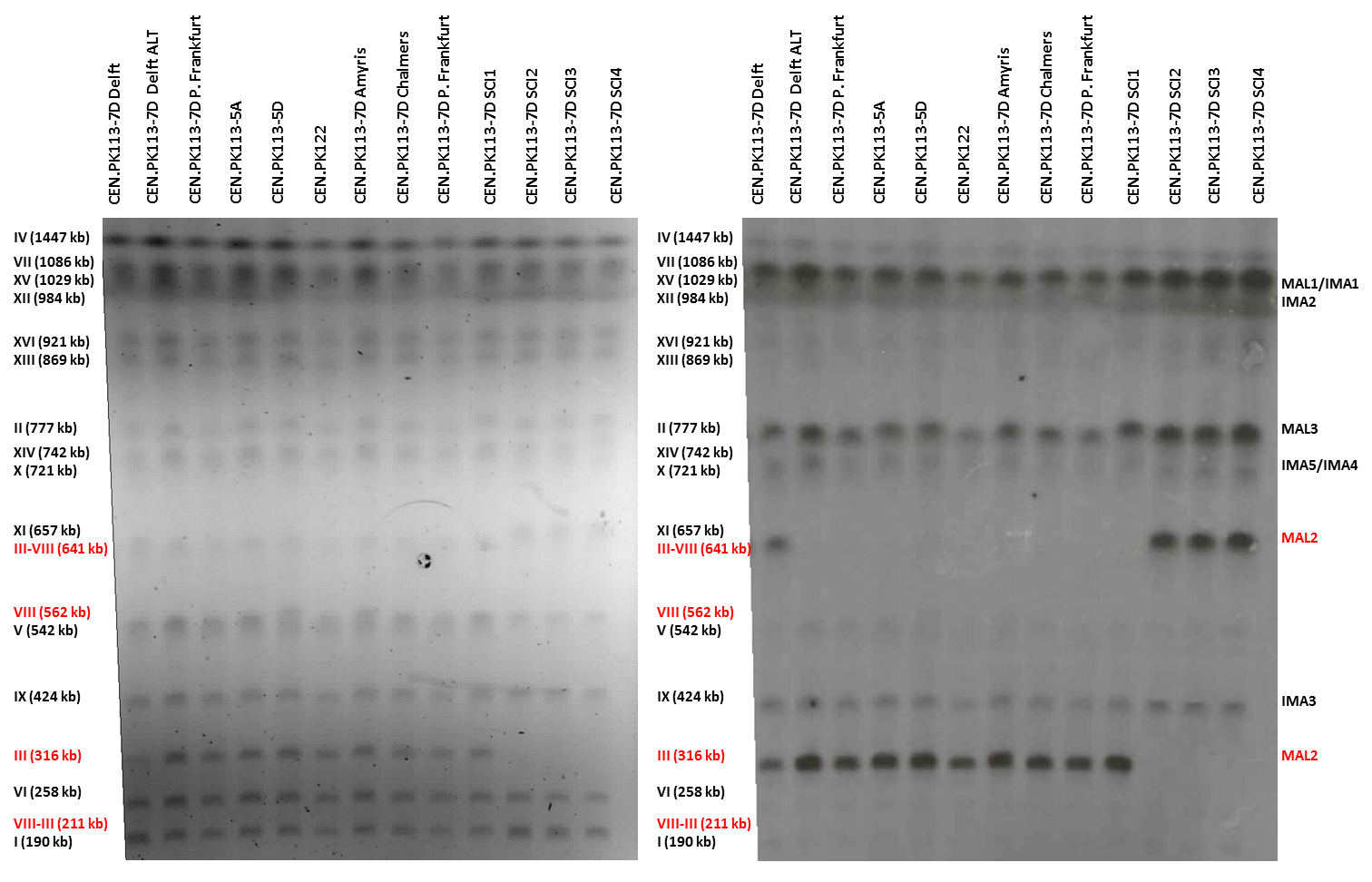


**Figure 1. Experimental validation of the heterogeneous population of CEN.PK113-7D cells.** Karyotypes and southern blots of stocks of CEN.PK113-7D of various origins and of some related strains to investigate the presence of a translocation between chromosomes III and VIII. The stocks used in each lane are shown above the gel and the chromosomes are indicated to the left of each image with their size as determined in this study between parenthesis. On the far right, the genes with which the probe of the southern blot are binding to are shown. The chromosomes and genes implicated in the III-VIII translocation are shown in red. (Left) Chromosomes separated by contour-clamped homogeneous electric field electrophoresis on a 1% agarose gel in 0.5X TBE buffer after staining with ethidium bromide and imaging under UV light. The recombined chromosomes III-VIII and VIII-III have a very similar length to chromosomes XI and I, respectively, making them difficult to distinguish. (Right) Southern blots performed on the same gel using a *MAL32* probe generated with primers GCAGAAGGGCAATCTTTGAAAGTG and AGCAGCAAACAGCGTCTTGTC (5’ to 3’). Besides the MAL2 locus, this probe also binds to the loci MAL3 and MAL1 due to near-perfect sequence similarity and to a lesser extent to various IMA genes with sequence similarity ranging between 60 and 70% due to the relatively low hybridization temperature of 55˚C.

## **Conclusion:**

The presence of a fourth MAL locus in CEN.PK113-7D Delft is in accordance with a translocation between chromosomes III and VIII. CEN.PK113-7D Delft has both original and recombined chromosome architectures for these chromosomes. Stocks obtained from other laboratories were shown not to have this translocation.
