# Supplementary material for: Nanopore sequencing enables near-complete de novo assembly of Saccharomyces cerevisiae reference strain CEN.PK113-7D
Source: FEMS Yeast Res. 2017 Sep 13;17(7):fox074. doi: 10.1093/femsyr/fox074 (PMC5812507; doi:10.1093/femsyr/fox074)
Supplement: Supplemental material — Supplementary data are available at FEMSYR online. [file fox074_supp.zip › Supplementary Figure S1 Read-length distributions of different MinION sequencing runs of the CEN.PK113-7D Delft and CEN.PK113-7D Frankfurt..docx]

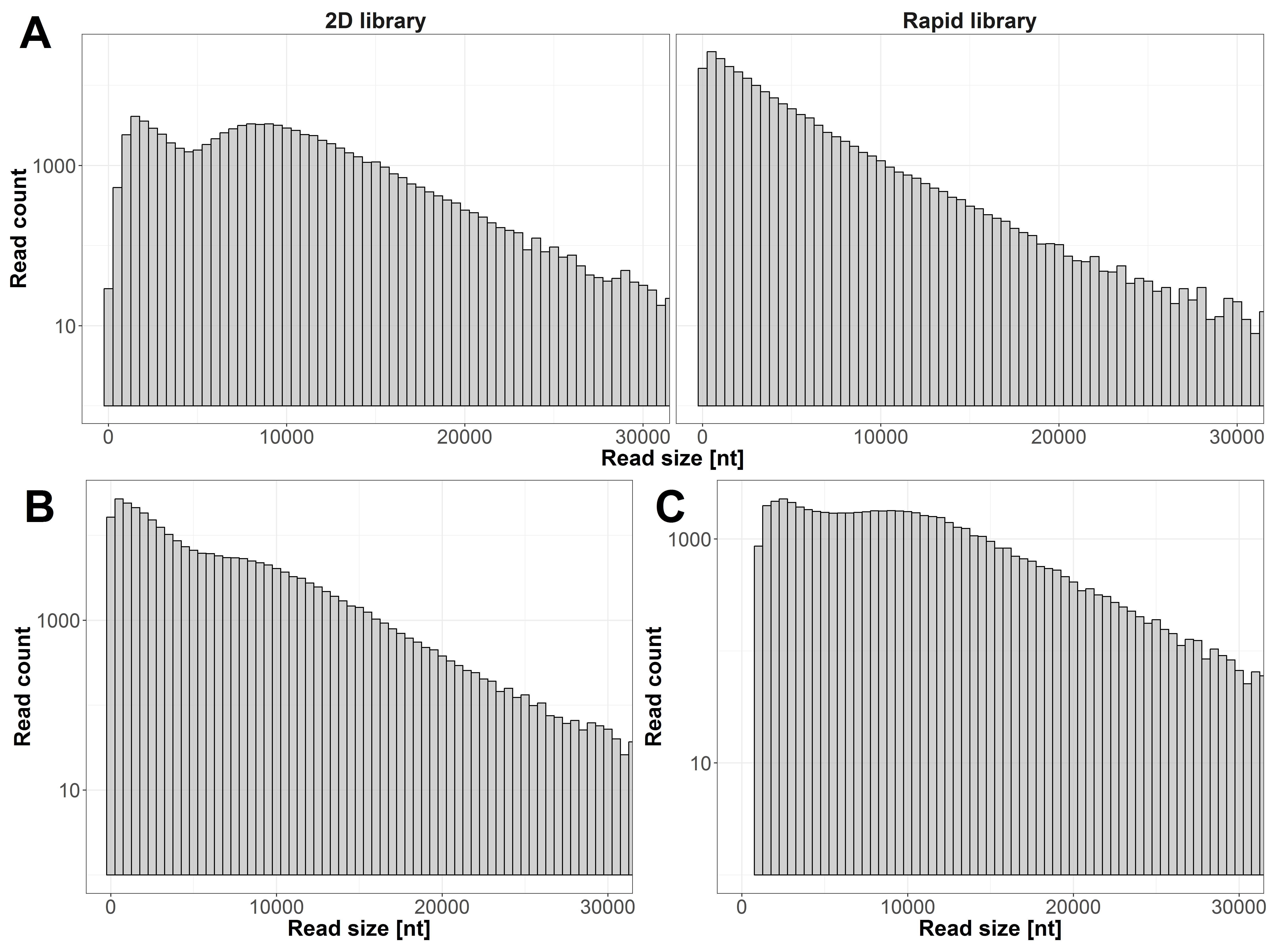
**Supplementary Figure S1. Read-length distributions of different MinION sequencing runs of the CEN.PK113-7D Delft and CEN.PK113-7D Frankfurt.** The figure shows the read length distributions of all MinION sequencing runs. In each plot, the X-axis represents read size capped at 30,000 bp and the Y-axis is read count in log10 scale. **(A)** reads from CEN.PK113-7D Delft acquired from 2D and Rapid library preparation methods on the left and right respectively. **(B)** combined reads of CEN.PK113-7D Delft in (A). **(C)** reads of CEN.PK113-7D Frankfurt acquired acquired from 2D library preparation methods.
