# Supplementary material for: Nanopore sequencing enables near-complete de novo assembly of Saccharomyces cerevisiae reference strain CEN.PK113-7D
Source: FEMS Yeast Res. 2017 Sep 13;17(7):fox074. doi: 10.1093/femsyr/fox074 (PMC5812507; doi:10.1093/femsyr/fox074)
Supplement: Supplemental material — Supplementary data are available at FEMSYR online. [file fox074_supp.zip › Supplementary Figure S2 Density histogram of error rates in the raw nanopore reads of CEN.PK113-7D Frankfurt and CEN.PK113-7D Delft..docx]

**
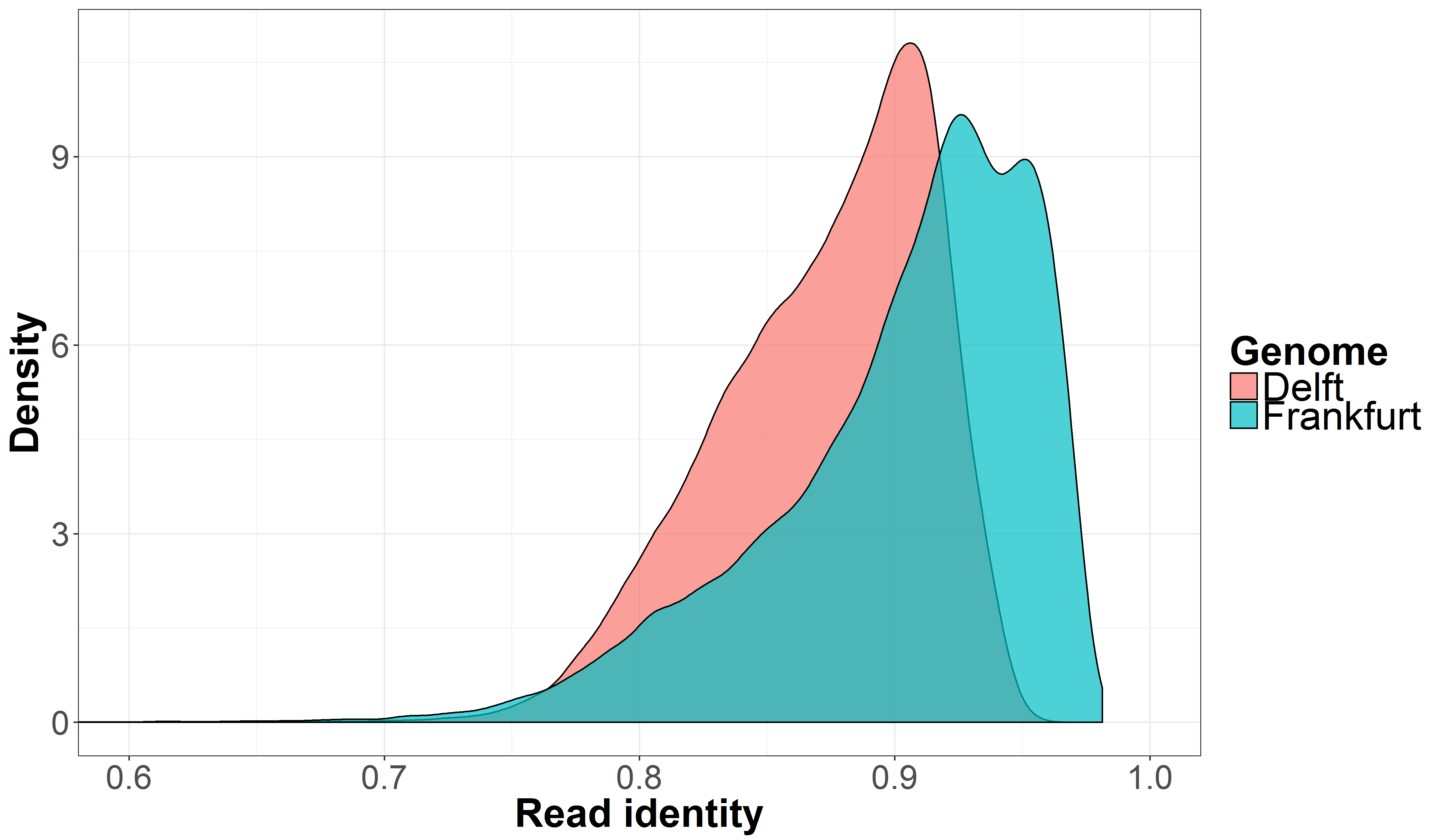
Supplementary Figure S2. Density histogram of error rates in the raw nanopore reads of CEN.PK113-7D Frankfurt and CEN.PK113-7D Delft.** The error rate of both read sets was determined by aligning them to the final CEN.PK113-7D Frankfurt assembly using Graphmap (Sovic *et al.* 2016). For CEN.PK113-7D Delft, a total of 110,909 reads were used: 55,735 template and 60,185 2D reads. For CEN.PK113-7D Frankfurt, a total of 36,544 were used: 16,077 template and 21,929 2D reads. The Delft data set contains a mixture of 2D and Rapid libraries while Frankfurt data set only contains a single 2D library. The mean read identify for CEN.PK113-7D Delft is 87% and CEN.PK113-7D Frankfurt is 90%.
