# Supplementary material for: Nanopore sequencing enables near-complete de novo assembly of Saccharomyces cerevisiae reference strain CEN.PK113-7D
Source: FEMS Yeast Res. 2017 Sep 13;17(7):fox074. doi: 10.1093/femsyr/fox074 (PMC5812507; doi:10.1093/femsyr/fox074)
Supplement: Supplemental material — Supplementary data are available at FEMSYR online. [file fox074_supp.zip › Supplementary Figure S3 Coverage across chromosome III contig before misassembly correction in the CEN.PK113-7D Frankfurt assembly..docx]

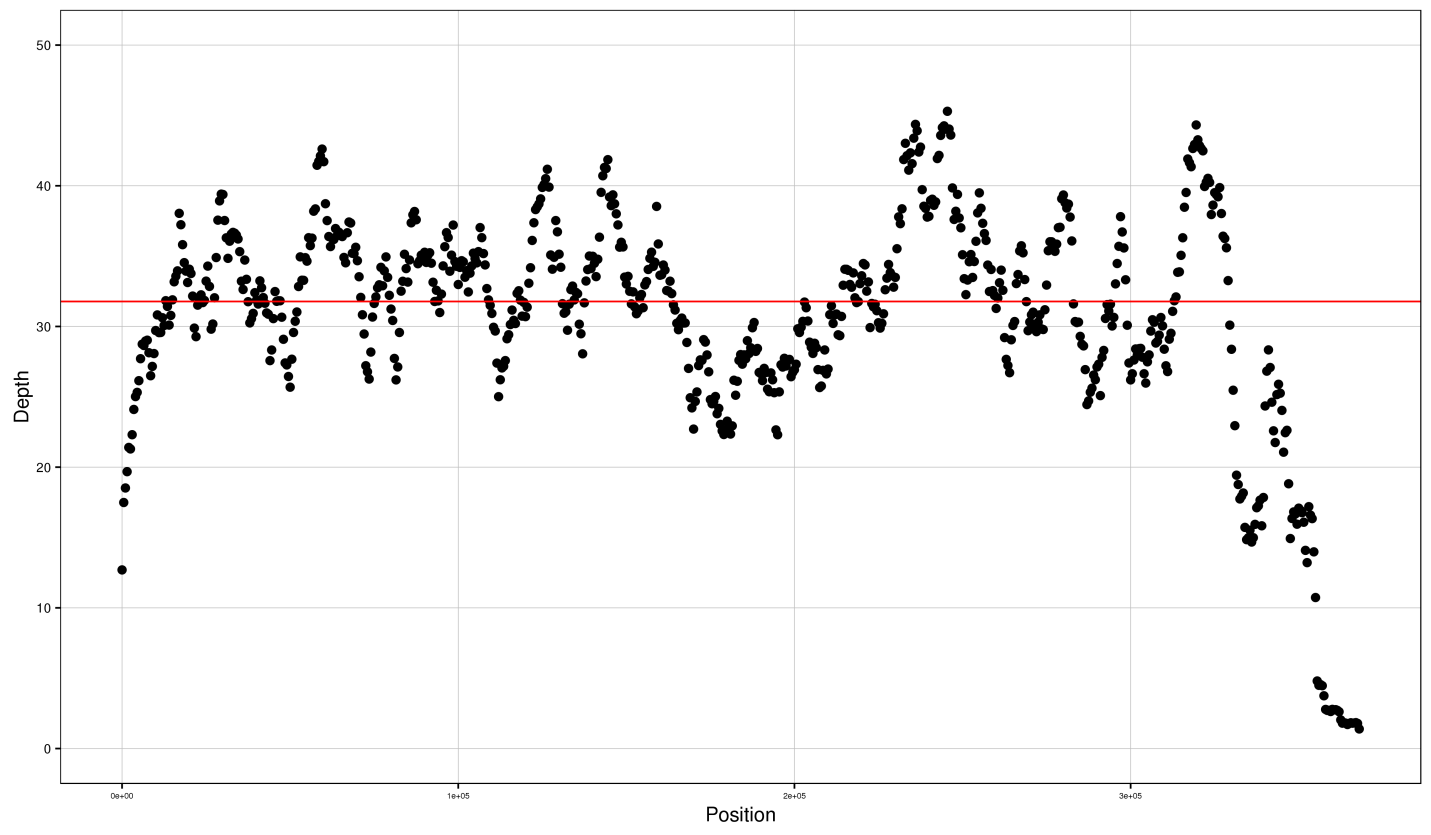


**Supplementary Figure S3. Coverage across chromosome III contig before misassembly correction in the CEN.PK113-7D Frankfurt assembly.** The figure shows the coverage plot of the initial contig corresponding to chromosome III in CEN.PK113-7D Frankfurt. The Y-axis shows the depth in coverage and X-axis is the position in the contig. Each dot represents the average coverage of non-overlapping windows of 500 nt based on nanopore read alignments of at least 6 Kbp in size and with a mapping quality of 10. The red line represents the calculated median coverage across the entire contig. The last 27 Kbp of the contig had very low coverage.
