# Supplementary material for: Nanopore sequencing enables near-complete de novo assembly of Saccharomyces cerevisiae reference strain CEN.PK113-7D
Source: FEMS Yeast Res. 2017 Sep 13;17(7):fox074. doi: 10.1093/femsyr/fox074 (PMC5812507; doi:10.1093/femsyr/fox074)
Supplement: Supplemental material — Supplementary data are available at FEMSYR online. [file fox074_supp.zip › Supplementary Figure S4 Alignment to the mitochondrial genome of S288C.docx]

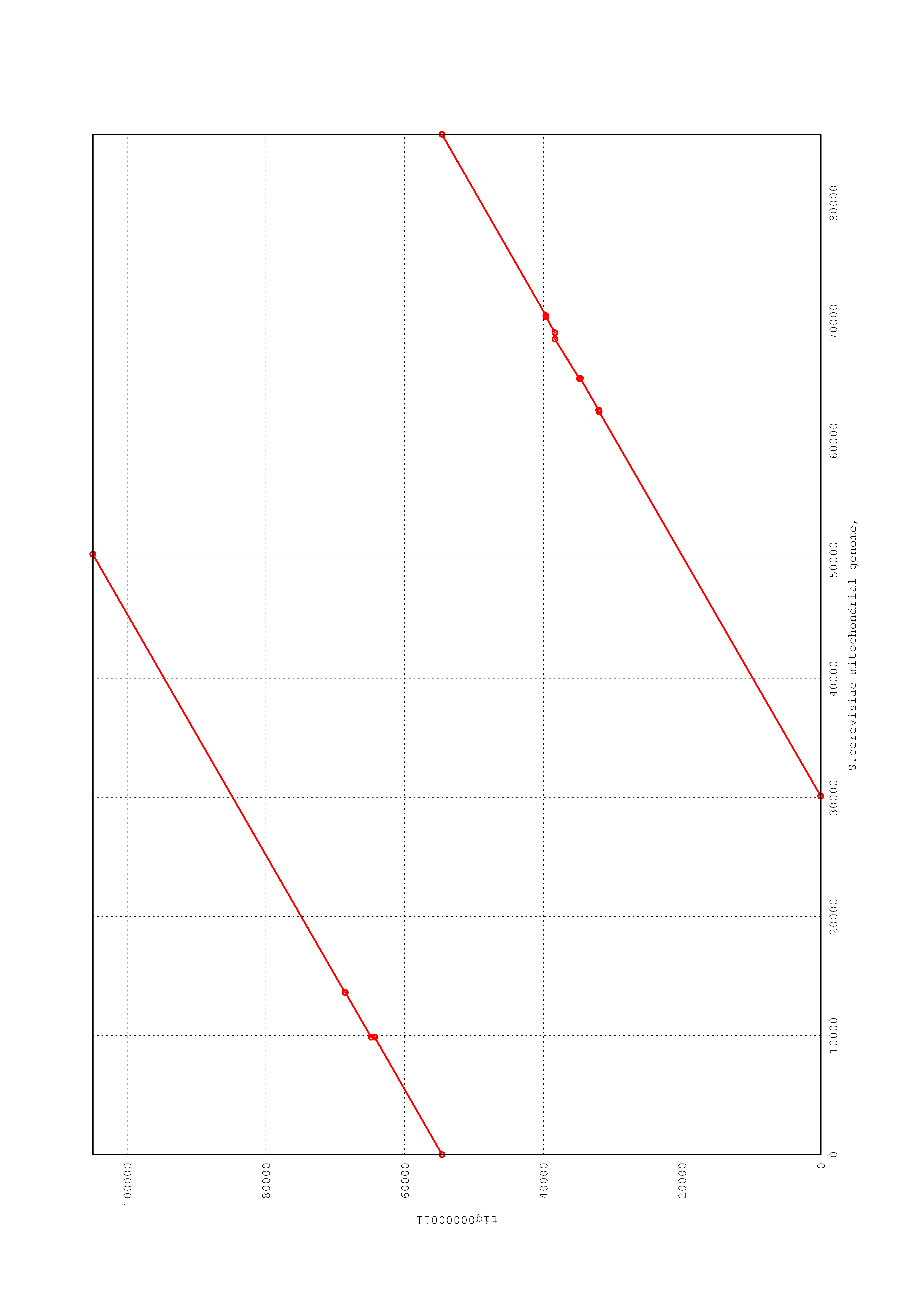


**Supplementary Figure S4. Alignment to the mitochondrial genome of S288C of the mitochondrial contig of the nanopore assembly of CEN.PK113-7D Frankfurt before misassembly correction.** The figure shows a mummer plot of the alignment of the contig to the mitochondrial genome in S288C. The Y-axis represents the contig corresponding to the mitochondrial genome in the initial assembly of the Frankfurt strain of CEN.PK113-7D and the X-axis is the S288C mitochondrial genome. The contig in CEN.PK113-7D had a size of 104 Kbp, while the S288C mitochondrial genome is only 86 Kbp. The CEN.PK113-7D aligned twice to positions 30,148 to 50,488, indicating this region was duplicated in the assembly.
