# Supplementary material for: Nanopore sequencing enables near-complete de novo assembly of Saccharomyces cerevisiae reference strain CEN.PK113-7D
Source: FEMS Yeast Res. 2017 Sep 13;17(7):fox074. doi: 10.1093/femsyr/fox074 (PMC5812507; doi:10.1093/femsyr/fox074)
Supplement: Supplemental material — Supplementary data are available at FEMSYR online. [file fox074_supp.zip › Supplementary Figure S5 Alignment of CEN.PK113-7D Delft to CEN.PK113-7D Frankfurt..docx]

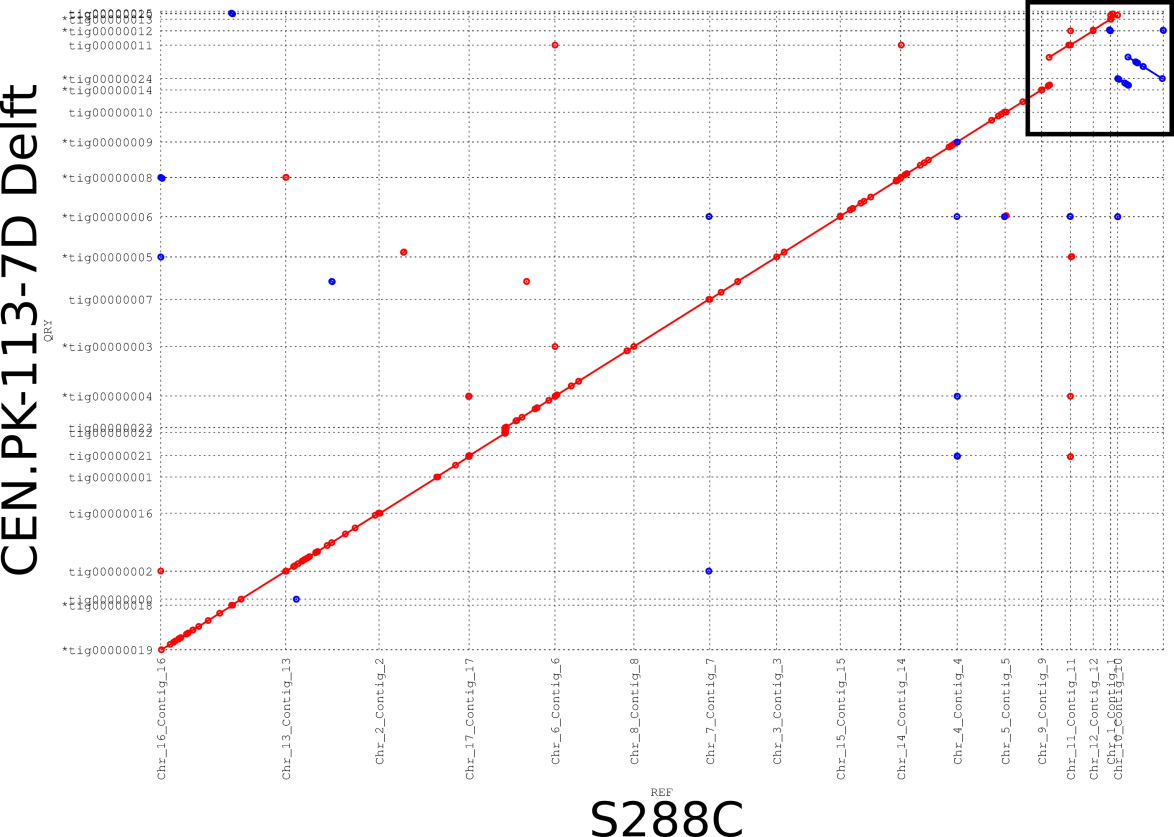


**Supplementary Figure S5. Alignment of CEN.PK113-7D Delft to CEN.PK113-7D Frankfurt.** The Y-axis corresponds to the nanopore assembly CEN.PK113-7D Delft while the X‑axis corresponds to the nanopore assembly CEN.PK113-7D Frankfurt after misassembly correction. The alignment shows a translocation between the contigs corresponding to chromosomes III and VIII (black box at the top-right corner of the figure) in CEN.PK113-7D Delft.
