# Supplementary material for: Nanopore sequencing enables near-complete de novo assembly of Saccharomyces cerevisiae reference strain CEN.PK113-7D
Source: FEMS Yeast Res. 2017 Sep 13;17(7):fox074. doi: 10.1093/femsyr/fox074 (PMC5812507; doi:10.1093/femsyr/fox074)
Supplement: Supplemental material — Supplementary data are available at FEMSYR online. [file fox074_supp.zip › Supplementary Table S1 Length of the contigs of the nanopore assembly of CEN.PK113-7D Delft and of the nanopore assembly of CEN.PK113-7D Frankfurt before misass.docx]

**Supplementary Table S1. Length of the contigs of the nanopore assembly of CEN.PK113-7D Delft and of the nanopore assembly of CEN.PK113-7D Frankfurt before misassembly correction.** CEN.PK113-7D Delft was sequenced with three flow cells using the R7.3 chemistry, while CEN.PK113-7D Frankfurt was sequenced on a single flow cell using the R9 chemistry. Both assemblies were obtained using Canu.

| **Delft** | | **Frankfurt** | |
| --- | --- | --- | --- |
| **Contig** | **Size (nt)** | **Contig** | **Size (nt)** |
| tig00000002 | 1086846 | tig00000015 | 1110333 |
| tig00000004 | 931717 | tig00000000 | 1073122 |
| tig00000003 | 884540 | tig00000020 | 960539 |
| tig00000019 | 837134 | tig00000005 | 939378 |
| tig00000007 | 796621 | tig00000007 | 902596 |
| tig00000005 | 755508 | tig00000006 | 797021 |
| tig00000006 | 736461 | tig00000001 | 756313 |
| tig00000016 | 680242 | tig00000022 | 719453 |
| tig00000008 | 666084 | tig00000017 | 671251 |
| tig00000024 | 631718 | tig00000023 | 596821 |
| tig00000023 | 590878 | tig00000003 | 571173 |
| tig00000009 | 559875 | tig00000009 | 544985 |
| tig00000000 | 527433 | tig00000018 | 535579 |
| tig00000021 | 435617 | tig00000004 | 435227 |
| tig00000010 | 417351 | tig00000010 | 424206 |
| tig00000001 | 401007 | tig00000008 | 365410 |
| tig00000011 | 267182 | tig00000012 | 269083 |
| tig00000012 | 216841 | tig00000013 | 206543 |
| tig00000014 | 212717 | tig00000011 | 104982 |
| tig00000018 | 111849 | tig00000021 | 38603 |
| tig00000013 | 100846 | tig00000016 | 31223 |
| tig00000022 | 92030 | **Total** | **12053841** |
| tig00000015 | 36456 |  |  |
| tig00000020 | 13133 |  |  |
| **Total** | **11990086** |  |  |
