# Supplementary material for: Nanopore sequencing enables near-complete de novo assembly of Saccharomyces cerevisiae reference strain CEN.PK113-7D
Source: FEMS Yeast Res. 2017 Sep 13;17(7):fox074. doi: 10.1093/femsyr/fox074 (PMC5812507; doi:10.1093/femsyr/fox074)
Supplement: Supplemental material — Supplementary data are available at FEMSYR online. [file fox074_supp.zip › Supplementary Table S2 Overview of the telomere caps in the nanopore assembly of CEN.PK113-7D Frankfurt..docx]

**Supplementary Table S2. Overview of the telomere caps in the nanopore assembly of CEN.PK113-7D Frankfurt.** For each chromosome, the assembled telomere caps are shown, and for chromosomes with missing telomere caps the last annotation and an estimation of the amount of missing sequence based on homology to S288C are shown. In total 6 telomeric caps were not assembled, resulting in an estimated 52 Kbp of missing sequence.

| **Chromosome** | **Telomere caps** | **Last annotation** | **Estimated distance to telomere** |
| --- | --- | --- | --- |
| CHR I | Right | YAR061W | 11 Kbp |
| CHR II | Both |  |  |
| CHR III | Both |  |  |
| CHR IV | Both |  |  |
| CHR V | Both |  |  |
| CHR VI | Left | YFR055W | 1 Kbp |
| CHR VII | Both |  |  |
| CHR VIII | Left | YHRCTy1-1 | 7 Kbp |
| CHR IX | Both |  |  |
| CHR X | None | YJL219W,YJR156C | 11 Kbp, 12 Kbp |
| CHR XI | Both |  |  |
| CHR XII | Both |  |  |
| CHR XII | Both |  |  |
| CHR XIV | Right | YNL334C-like | 10 Kbp |
| CHR XV | Both |  |  |
| CHR XVI | Both |  |  |
