# Supplementary material for: Nanopore sequencing enables near-complete de novo assembly of Saccharomyces cerevisiae reference strain CEN.PK113-7D
Source: FEMS Yeast Res. 2017 Sep 13;17(7):fox074. doi: 10.1093/femsyr/fox074 (PMC5812507; doi:10.1093/femsyr/fox074)
Supplement: Supplemental material — Supplementary data are available at FEMSYR online. [file fox074_supp.zip › Supplementary Table S3 Lengths and gaps present of the contigs of the definitive nanopore assembly of CEN.PK113-7D Frankfurt..docx]

**Supplementary Table S3. Lengths and gaps present of the contigs of the definitive nanopore assembly of CEN.PK113-7D Frankfurt.** The final assembly metrics for the genome of CEN.PK113-7D Frankfurt after misassembly correction, manual scaffolding, and error correction with nanopore and Illumina data. Only chromosome XII has an unclosed gap corresponding to the ribosomal DNA locus (*RDN1*)—a region composed of repetitive sequences estimated to be more than 1 Mbp long (Venema and Tollervey 1999).

| **Chromosome** | **Size (nt)** | **Gaps** |  |
| --- | --- | --- | --- |
| CHR IV | 1504372 | 0 |  |
| CHR VII | 1123019 | 0 |  |
| CHR XV | 1085503 | 0 |  |
| CHR XII | 1032974 | 1 (RDN1 locus) | |
| CHR XVI | 950369 | 0 |  |
| CHR XIII | 912802 | 0 |  |
| CHR II | 806478 | 0 |  |
| CHR XIV | 765137 | 0 |  |
| CHR X | 727362 | 0 |  |
| CHR XI | 678882 | 0 |  |
| CHR V | 577587 | 0 |  |
| CHR VIII | 550966 | 0 |  |
| CHR IX | 440269 | 0 |  |
| CHR III | 348163 | 0 |  |
| CHR VI | 272224 | 0 |  |
| CHR I | 208852 | 0 |  |
| Mitochondria | 86616 | 0 |  |
| Unplaced subtelomeric fragment 1 | 38649 | 0 |  |
| Unplaced subtelomeric fragment 2 | 31222 | 0 |  |
| **Total** | **12147760** |  |  |
